# Mechanisms of Temperature‐Dependent Hysteresis in Freestanding BaTiO_3_/MoS_2_ Heterostructures

**DOI:** 10.1002/advs.77639

**Published:** 2026-09-08

**Authors:** Thomas Pucher, Federico Mompeán, Víctor Rouco, Carlos León, Jacobo Santamaría, Carmen Munuera, Mar García‐Hernández, Andres Castellanos‐Gomez

**Affiliations:** ^1^ 2D Foundry Research Group Instituto de Ciencia de Materiales de Madrid (ICMM‐CSIC) Madrid Spain; ^2^ Unidad Asociada UCM/CSIC “Laboratorio de Heteroestructuras Con Aplicación En Spintrónica” Madrid Spain; ^3^ GFMC Departamento de Física de Materiales Facultad de Física Universidad Complutense Madrid Spain

**Keywords:** 2D materials, complex oxides, ferroelectricity, freestanding, TMDs

## Abstract

Freestanding ferroelectric oxides integrated with 2D semiconductors offer a platform for reconfigurable electronic functionalities beyond conventional dielectric gating. However, once released from epitaxial constraint and transferred directly onto metallic gates, the BaTiO_3_ (BTO) membranes under study preferentially adopt an in‐plane polarization configuration, suppressing out‐of‐plane ferroelectric coupling at room temperature. Here, we demonstrate that single‐layer MoS_2_ field‐effect transistors gated by 25 nm freestanding BTO exhibit excellent electrostatic control at room temperature, with subthreshold swings down to 85 mV dec^−^^1^ and high on/off ratios, yet negligible hysteresis, highlighting the effectiveness of depolarized BTO as an ultrahigh‐κ dielectric. Upon cooling, robust counter‐clockwise ferroelectric hysteresis emerges, with memory windows of ∼0.4 V, stable over 500 cycles and retention times exceeding 10^5^ s. Temperature‐dependent x‐ray diffraction reveals structural signatures consistent with the bulk phase transitions sequence of BTO and confirms a predominant in‐plane lattice orientation near room temperature. The combined electrical and structural analysis indicates that phase‐dependent polarization anisotropy, together with domain‐wall dynamics and interfacial screening processes, stabilizes an out‐of‐plane polarization component at low temperature in the rhombohedral and orthorhombic phases.

## Graphical abstract

Freestanding ferroelectric BaTiO_3_ membranes are combined with monolayer MoS_2_ into 2D field‐effect transistors. At room temperature they provide ultrahigh‐κ gating with steep subthreshold swing and minimal hysteresis. Upon cooling, strong ferroelectric switching produces two stable memory states with long retention times. X‐ray diffraction measurements reveal the temperature‐dependent connection between phase transitions and electric hysteresis.

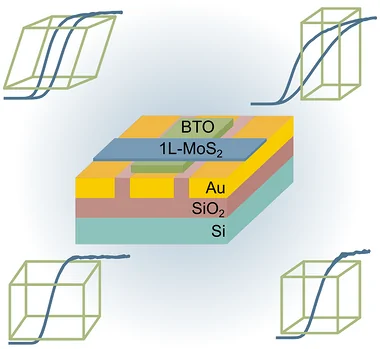

## 1 Introduction

The integration of complex oxides with 2D semiconductors has emerged as a powerful strategy to engineer electronic and optical functionalities that are difficult to achieve with conventional dielectric stacks [1, 2]. In particular, the combination of transition‐metal dichalcogenides (TMDs) with perovskite oxides such as BaTiO_3_ (BTO) enables access to ultrahigh‐κ screening, large built‐in polarization fields, and reconfigurable electrostatic environments [3, 4, 5], all without relying on direct oxide growth on the atomically thin semiconductor. Recent advances in the synthesis of freestanding perovskite membranes have opened a promising route to assemble such heterostructures through low‐impact, transfer‐based processes that preserve the integrity of the 2D channel [6, 7, 8, 9, 10].

Recent structural studies of freestanding BTO membranes have shown that, once released from epitaxial constraint, they relax into ferroelectric states with polarization predominantly confined to the plane of the membrane, as the absence of mechanical clamping suppress the stability of an out‐of‐plane dipole at room temperature [11]. Note that other recent studies on different sets of freestanding BTO membranes have also observed a relaxation of the strain along the out‐of‐plane axis after releasing the membrane from the epitaxial substrate while still maintaining the out‐of‐plane polarization, suggesting that the exact growth and releasing process can lead to very different sets of samples exhibiting a variety of intrinsic properties [12, 13]. However, the membrane polarization may be strongly influenced by the electrostatic and dielectric environments when stacked in a heterostructure conforming a device. Gate‐tunable photoluminescence measurements of MoS_2_ flakes transferred onto the BTO films deposited directly onto a metal gate (Au) demonstrate that the membrane is unable to maintain a stable out‐of‐plane polarization at room temperature, again pointing to a naturally depolarized, intrinsic in‐plane configuration in our samples [4]. By contrast, introducing an insulating layer between the metal gate and the BTO restores robust out‐of‐plane polarization even at room temperature and decreases the leakage currents. This behavior is reminiscent to many ferroelectric transistors employing a metal‐ferroelectric‐insulator‐semiconductor (MFIS) stack, in which an interfacial dielectric layer is inserted to passivate the semiconductor surface chemistry and help control depolarization fields and stabilize out‐of‐plane polarization [14]. However, the inclusion of an insulating layer necessarily places the ferroelectric in series with a lower‐κ dielectric, reducing the effective gate capacitance and preventing full exploitation of the intrinsically ultrahigh permittivity of BTO (i.e. a fundamental trade‐off in device design) [3]. Achieving stable out‐of‐plane polarization in freestanding BTO without a parallel dielectric layer would therefore remove this series‐capacitance penalty and allow devices to fully exploit the exceptional permittivity of BTO.

Here we address these questions by investigating single‐layer MoS_2_ transistors gated with freestanding BTO membranes transferred directly onto a gold gate electrode. At room temperature, the devices exhibit excellent electrostatics (i.e., subthreshold swings down to 85 mV dec^−^^1^) yet display negligible hysteresis, consistent with the expected depolarization of the out‐of‐plane polarization component. Interestingly, temperature‐dependent measurements reveal a striking re‐emergence of ferroelectric hysteresis at low temperature. This observation is consistent with depolarizing fields and interfacial screening processes being thermally activated: at room temperature, incomplete screening of polarization charges by the metallic gate and semiconducting channel [15], combined with mobile adsorbates and defect‑mediated screening charges at the oxide interfaces [16], lead to residual depolarizing fields and back‑switching that suppress a net out‑of‑plane polarization component. At low temperature, the mobility of these interfacial species and traps is strongly reduced and domain walls become more strongly pinned [17], so that an out‑of‑plane polarization component can remain stable over a longer timescale and imprint a robust hysteresis in the MoS_2_ transfer characteristics. Furthermore, the freestanding BTO membranes show changes in crystal symmetry at low temperature, probed with x‐ray diffraction (XRD) at different temperatures, that can also favor the out‐of‐plane polarization. As a result, the same membrane transitions from a depolarized high‐κ dielectric at room temperature to a remanent ferroelectric at low temperature.

## 2 Results and Discussion

### 2.1 Device Fabrication

A buried three‐electrode design was prepared by maskless lithography and a subsequent etching of the SiO_2_ layer (50 nm depth), followed by metal deposition of 5 nm Cr and 45 nm Au, resulting in a planar surface of Au and SiO_2_ [4, 18, 19].

Freestanding BTO membranes were obtained by releasing epitaxial BTO films from their growth substrate following the procedure reported in Ref. [11]. In brief, 25 nm‐thick BTO layers were grown on LSMO‐buffered SrTiO_3_, where the LSMO acts as a sacrificial layer. A thin film of polypropylene carbonate (PPC) was spin‐coated onto the BTO surface and cured, after which the sample was attached to a PDMS stamp. The PPC/PDMS‐supported film was then immersed in a selective KI + HCl etchant, which dissolves the LSMO layer and causes the BTO film to delaminate cleanly from the substrate after several days of etching. Once released, the free‐standing BTO membrane, still supported by the PPC/PDMS stack, was rinsed in deionized water and could be deterministically transferred onto a SiO_2_/Si handling substrate (which acts as a reservoir of BTO sheets) for further device assembly.

**FIGURE 1:**
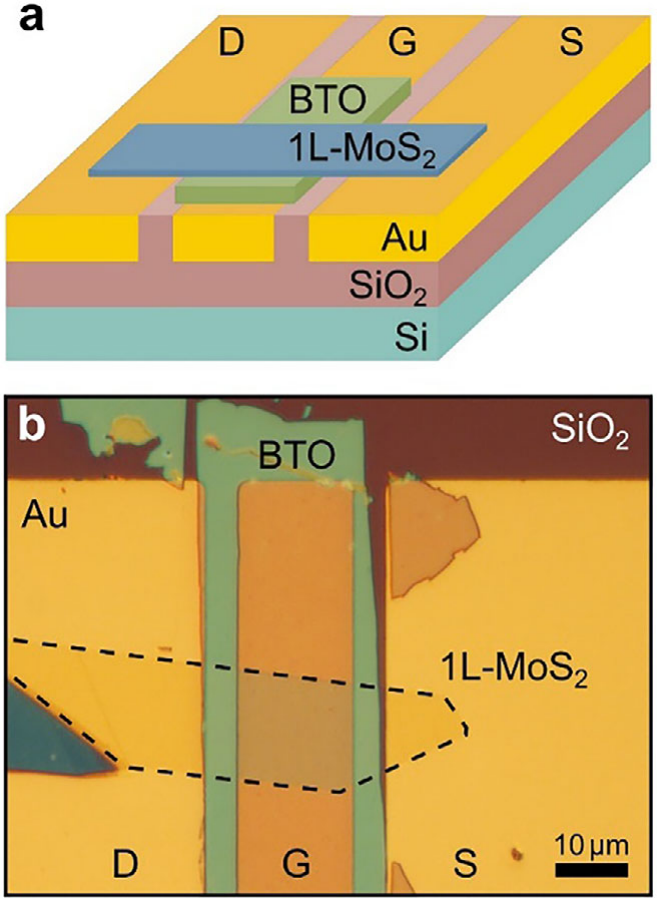
Structure and fabrication of BTO‐based field‐effect transistors. (a) Schematic of the transistor. The electrodes are buried inside the SiO_2_ layer of the substrate, creating a planar interface. The BTO (25 nm) is transferred on top of the gate electrode (G) and the monolayer MoS_2_ is transferred on top of the BTO, composing the semiconducting channel by bridging the drain (D) and source (S) electrodes. (b) Microscope image of the fabricated device. The channel length is 25 µm and the channel width is 14.5 µm.

To fabricate the FETs, a selected BTO flake (with right dimensions to fully cover the gate electrode) was picked up using a PVC/PDMS dry‐transfer stamp and placed onto the gate electrode [20]. Later, monolayer MoS_2_ flakes were mechanically exfoliated onto PDMS, identified using transmission‐mode optical microscopy, and their thickness verified by differential reflectance spectroscopy [21, 22, 23]. The chosen MoS_2_ flake was then dry‐transferred onto the BTO membrane [24], bridging the source and drain contacts to complete the channel. An illustration and an optical image of a representative device (25 nm BTO and monolayer MoS_2_) are shown in Figure 1.

### 2.2 BTO/MoS_2_ FET Output Characteristics

**FIGURE 2:**
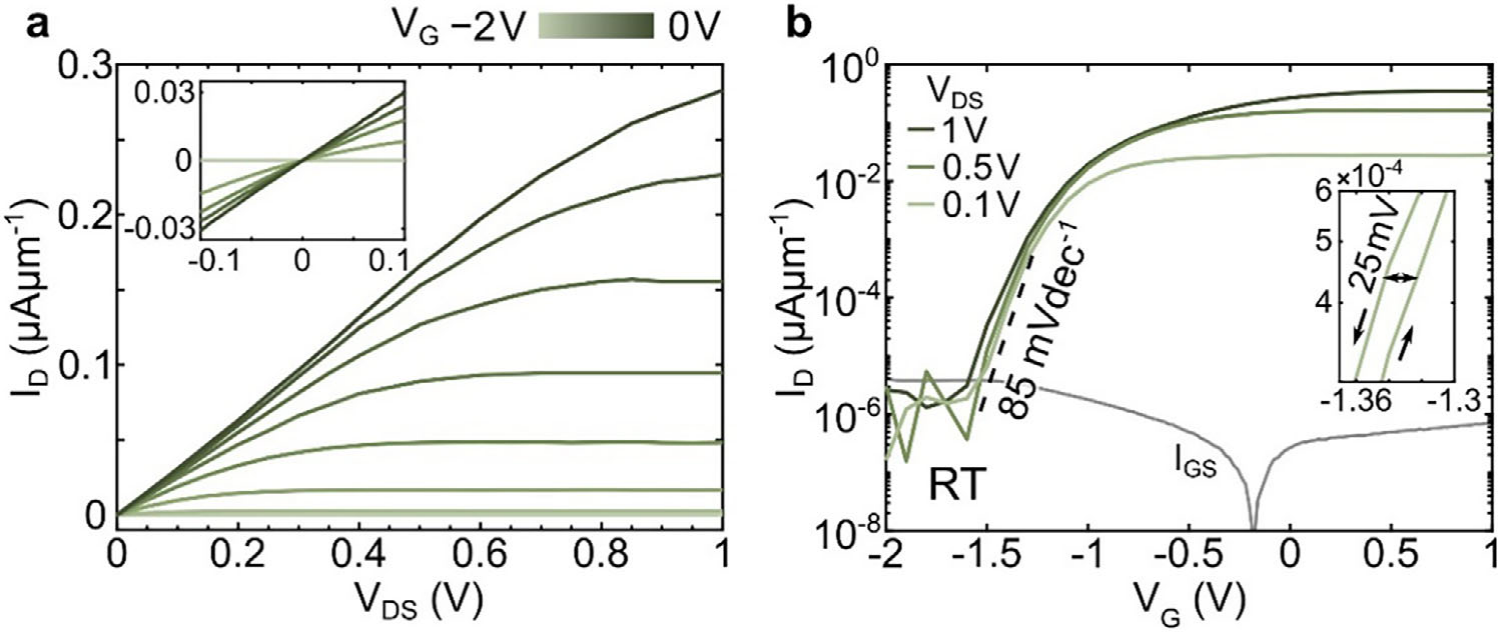
BTO/1L‐MoS_2_ FET figures of merit. (a) Output characteristics of the device shown in Figure 1b, illustrating saturating *I–V*s. (Inset) Linear *I–V*s around the zero‐voltage region. The gate voltage was swept between −2 and 0 V. (b) Transfer curves at different bias situations (*V*_DS_ = 0.1, 0.5, and 1 V) at room temperature, including the leakage current (*I*_GS_). The inset shows a magnified view of the hysteresis between forward and backward sweeps for *V*_DS_ = 0.1 V, indicating minimal counter‐clockwise hysteresis.

Figure 2 shows the room‐temperature electrical measurements of a single‐layer MoS_2_ transistor with a 25 nm thick BTO gate dielectric. Figure 2a illustrates the output curves of the transistor with a horizontal saturation current and linear current‐voltage curves (*I–Vs*) around the zero‐voltage regime (inset), suggesting ohmic contacts and low Schottky barriers. The transfer curves for different source‐drain biases, together with the gate leakage current are plotted in Figure 2b. The transfer curves show steep subthreshold behavior, with a minimal subthreshold swing (*SS*) of 85 mV dec^−1^ and a threshold voltage *V*_T_ of about −1.2 V. The three transfer traces are nearly parallel in subthreshold, indicating modest drain‐induced barrier lowering (*DIBL*). The current on/off ratio is in the order of 10^5^–10^6^, on‐state currents are high in the order of 10^−1^–10^0^ µA µm^−1^ and the counter‐clockwise hysteresis minimal. The inset illustrates a hysteresis of 25 mV for V_DS_ = 0.1 V. These measurements are taken at room temperature in vacuum conditions (10^−6^ mbar), right after a thermal annealing step at 200°C in the same vacuum conditions to improve the gold‐MoS_2_ contacts and remove adsorbates.

The characterization of the transistors clearly shows an improved device performance when solely using BTO as the gate dielectric, compared to previous results with SiO_2_/BTO gate stacks [3]. The advantages among others include steeper *SS*, higher on currents and lower threshold voltages. However, what disappeared is the prominent ferroelectric hysteresis, which was present in the transfer curves when including a SiO_2_ layer between the BTO and the gate electrode. Other works have attributed a depolarization at room temperature to the predominant in‐plane polarization within freestanding BTO films [4, 11]. In fact, many ferroelectric transistors (FeFETs) nowadays rely on a metal‐ferroelectric‐insulator‐semiconductor structure to keep a stable polarization during device operation [25, 26]. In our device geometry, the freestanding BTO membrane is directly interfaced with a metallic gate electrode on one side and the MoS_2_ channel on the other side. Under these electrostatic boundary conditions, finite screening lengths in the electrodes and semiconducting channel, together with the absence of a dedicated blocking layer, can give rise to residual depolarizing fields that favor in‑plane polarization and multidomain configurations, making a robust out‑of‑plane memory window difficult to observe at room temperature. Related work on free‑standing PZT membranes has shown that the electrostatic and dielectric environment provided by the base layer strongly influences ferroelectric properties [27].

Notably, our previous work Ref. [4] also demonstrated, through gate‐tunable photoluminescence (PL) measurements of MoS_2_ gated through a BTO dielectric, that BTO directly on gold electrodes does not retain out‐of‐plane polarization, further supporting the conclusion that the polarization is predominantly in‐plane under these conditions. Even in the absence of out‐of‐plane ferroelectric polarization, the device benefits from the ultrahigh‐κ response of BTO (*ε*_r_ ∼ 4000). The resulting gate capacitance provides far stronger electrostatic control than achievable with SiO_2_, yielding sharp subthreshold slopes, high on‐currents, and low threshold voltages. Thus, the depolarized BTO membrane still acts as an outstanding ultra‐high‐κ dielectric at room temperature. Although our transistor cannot electrically outperform similar devices realized with other freestanding ferroelectric oxides like HZO [28, 29] or PZT [30], electronic parameters, such as SS are similar to other reports on BTO‐based FeFETs [31]. A recent study has shown that stable room‐temperature memory windows with BTO‐gated transistors can be realized via a miniaturized dielectric‐channel design fabrication [32].

### 2.3 Low Temperature Output Characteristics

**FIGURE 3:**
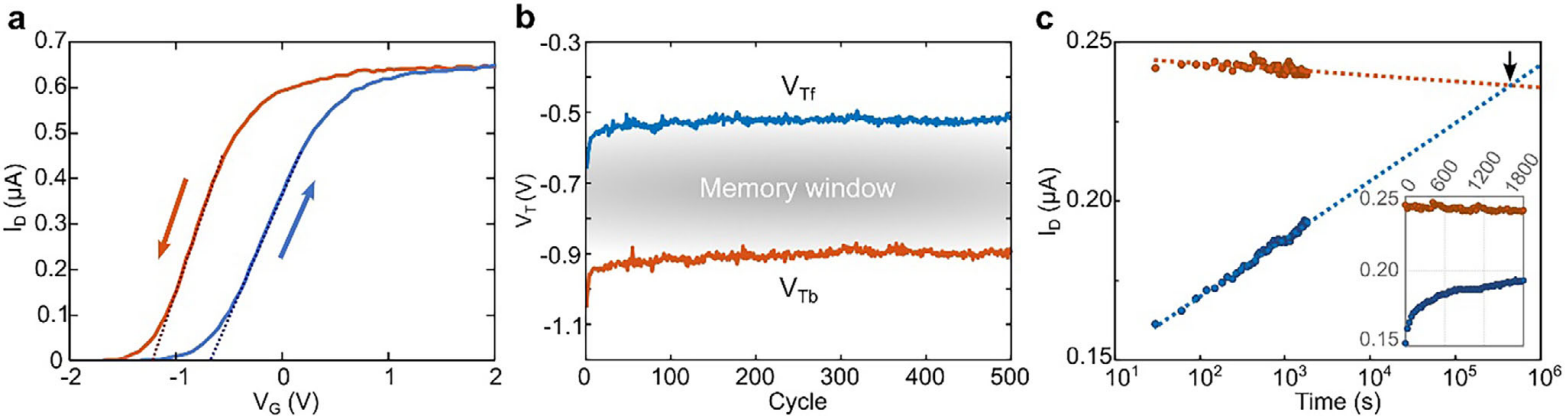
BTO/1L‐MoS_2_ FET 10 characterization. (a) Transfer curve at *V*_DS_ = 0.5 V measured at 10 K showing a clear memory window between forward (blue) and backward (red) sweeps. Dotted lines mark the threshold voltages (*V*_T_) extracted for each gate‐voltage sweep. (b) Evolution of the threshold voltages over 500 transfer curves at 10 K, demonstrating a stable and well‐defined memory window (~0.4 V). (c) Retention measurements (*V*_DS_ = 0.5 V) at 10 K showing the stability of the up and down polarization states in the BTO layer following the application of ± 4 V gate‐voltage pulses with a duration of 1s.

To evaluate whether out‐of‐plane ferroelectric polarization can stabilize in freestanding BTO despite its tendency toward in‐plane orientation at room temperature, we performed electrical measurements at 10 K, where bulk BTO is in its rhombohedral ferroelectric phase and thermal activation of depolarizing mechanisms is strongly suppressed. Figure 3 shows the transfer characteristics of the BTO/1L‐MoS_2_ transistor at 10 K. A clear counter‐clockwise hysteresis loop emerges, with well‐separated forward and backward traces that define a robust memory window. The loop maintains its shape across different sweep rates, confirming its ferroelectric origin rather than traps or slow charge relaxation (Figure S2).

The hysteresis magnitude (memory window of ~0.4 V) at 10 K is significantly larger than at room temperature, indicating that reduced thermal activation and frozen screening charges allow the out‐of‐plane component of the polarization to stabilize in the freestanding BTO membrane. These conditions suppress the mobile charge compensation that normally depolarizes the membrane at higher temperatures, enabling the ferroelectric dipole to couple effectively to the MoS_2_ channel.

The endurance of the memory window was tested by performing a series of cyclic forward‐backward transfer sweeps and monitoring the two corresponding threshold voltages *V*_T_ (Figure 3b). After 500 cycles, the forward and backward threshold voltages remain well separated, with negligible drift of either state. The persistence of the memory window through repeated cycling confirms that the device operates as a reliable ferroelectric FET at low temperature, with minimal wake‐up or fatigue effects. See the Supporting Information for the evolution of the *SS* depending on the cycle number.

The stability of the polarization states was evaluated through gate‐bias retention tests using ±4 V programming pulses (Figure 3c). Each state is measured by first applying the corresponding gate programming pulse for 1 s and then tracking the drain‐current *I*_D_ at a read‐out voltage of *V*_DS_ = 0.5 V for 30 min at *V*_G_ = 0 V. The two remanent states produce distinct and reproducible drain‐current levels that remain stable over at least ∼30 min of continuous monitoring. The extrapolated retention time from the logarithmic decay fits is 5 × 10^5^ s (∼5.6 days) at 10 K, demonstrating that the polarization is not only switchable but also long‐lived under cryogenic conditions.

Together, these measurements demonstrate that at 10 K the freestanding BTO membrane develops a stable out‐of‐plane ferroelectric polarization that is strongly coupled to the MoS_2_ channel. This behavior contrasts sharply with room temperature, where depolarization mechanisms suppress hysteresis, and highlights temperature as an effective knob for switching the device between a high‐*κ* transistor regime (RT) and a ferroelectric memory regime (low T).

### 2.4 Phase Transitions in Freestanding BTO

While the low‐temperature ferroelectric hysteresis can be rationalized by reduced thermal activation of interfacial screening charges and enhanced domain‐wall pinning, BTO is also known to undergo a sequence of crystallographic phase transitions, each associated with changes in lattice anisotropy and polarization orientation [33]. To assess whether such structural effects could provide an additional, or complementary, mechanism for stabilizing the observed ferroelectric hysteresis in freestanding membranes, we performed temperature‐dependent x‐ray diffraction (XRD) measurements on the BTO films (with 25 nm thickness). As mentioned above, BTO exhibits a well‐known sequence of structural phase transitions in bulk, each associated with a distinct symmetry and preferred polarization direction: rhombohedral (T < 180 K), orthorhombic (180–278 K), tetragonal (278–393 K), and cubic (T > 393 K) [33, 34]. In the rhombohedral phase, the spontaneous polarization lies close to ⟨111⟩‐type directions, while in the orthorhombic phase it aligns close to ⟨110⟩, and in the tetragonal phase it is oriented along ⟨001⟩. Note that the phase diagram in freestanding membranes may differ from that of the bulk, similarly to the case for as grown epitaxial thin films.

**FIGURE 4:**
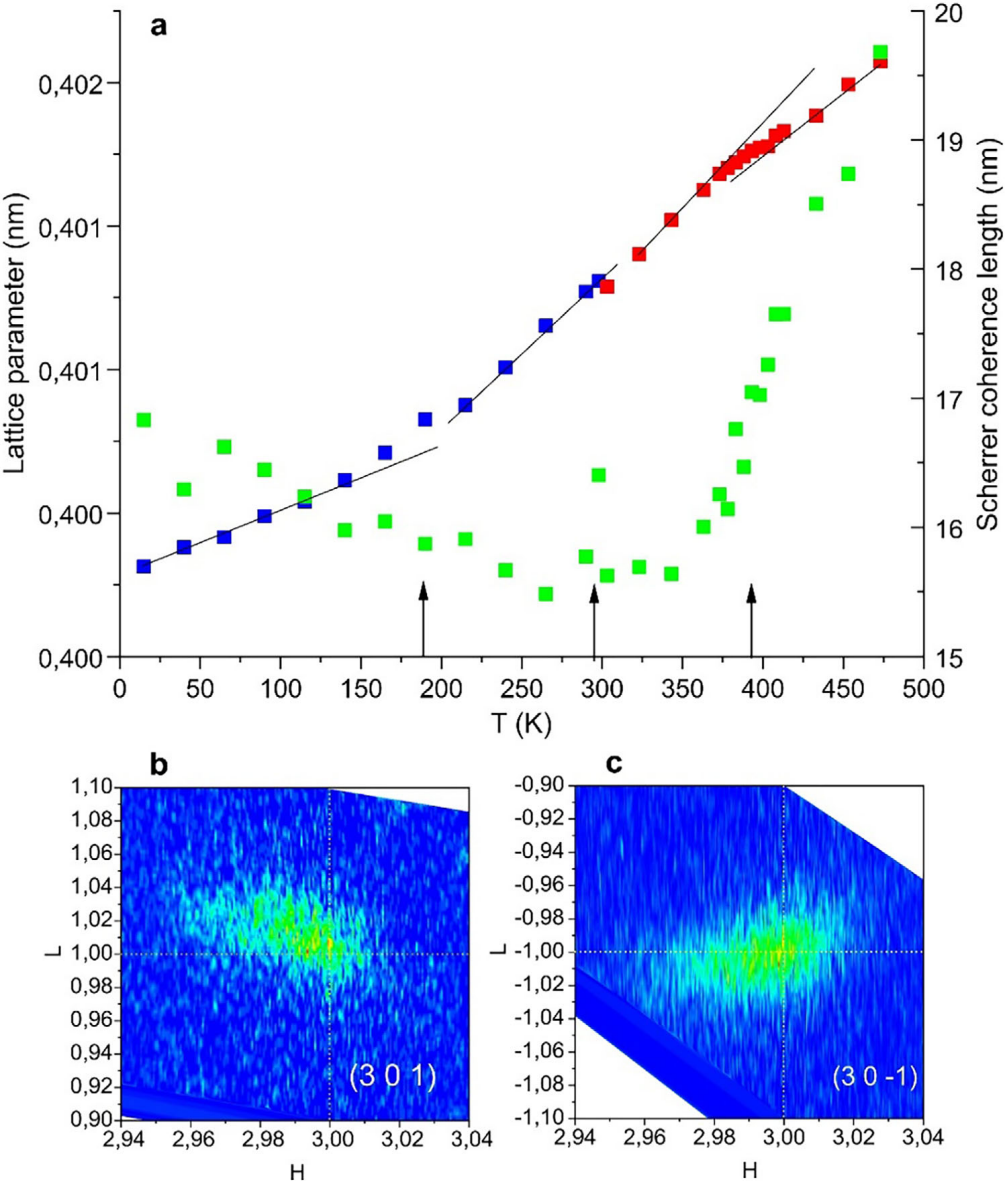
Structural characterization of freestanding BTO membranes. (a) Temperature dependence of the out‐of‐plane lattice parameter extracted from θ–2θ x‐ray diffraction scans (left axis, red/blue symbols) and the corresponding coherence length estimated from the diffraction peak width using the Scherrer equation (right axis, green symbols). Vertical arrows indicate temperatures at which changes in the slope of the lattice parameter and coherence length are observed, consistent with the vicinity of the rhombohedral–orthorhombic, orthorhombic–tetragonal, and tetragonal–cubic phase transitions of bulk BaTiO_3_. (b,c) Reciprocal‐space maps of the asymmetric (301) reflection measured at room temperature in the positive and negative diffractometer configurations by reference to tetragonal bulk BTO unit cell (Space Group P4mn with a = b = 0.3994 nm and c = 0.4038 nm), respectively. The maximum diffracted intensity is centered near the expected reciprocal‐space position for a tetragonal lattice with its elongated *c*‐axis oriented parallel to the membrane surface, indicating that the majority of the unit cells are arranged with their spontaneous polarization predominantly in‐plane. Weaker intensity tails suggest the presence of a minor fraction of differently oriented domains. Together, these measurements provide a structural framework for understanding the temperature‐dependent stabilization of ferroelectric coupling in the BTO/MoS_2_ transistors.

Near room temperature, the interesting regions for the BTO membrane XRD analysis are the broad peaks found near 2θ = 22.4 and 45.4 degrees. By reference to bulk BTO in the tetragonal phase (Space Group P4mn with a = b = 0.3994 nm and c = 0.4038 nm), these peaks fall respectively in the positions corresponding to (100) and (200) reflections. A similar analysis can be done with the θ–2θ scans collected as a function of temperature and Figure 4a summarizes the temperature dependence of the lattice spacing for BTO derived from the peak positions in the 2θ = 45.4 degree regions. Across the entire temperature range investigated, the measured lattice spacing remains smaller than the bulk tetragonal *c*‐axis parameter of BTO at room temperature (c ≈ 0.4038 nm) [35]. Assuming the bulk BTO phase diagram as a reference [33, 34], this observation indicates that the θ–2θ scans predominantly probe lattice parameters corresponding to the shorter axes (a or b), rather than the elongated *c*‐axis. This result implies that, at room temperature, the majority of unit cells are oriented with their long axis lying parallel to the sample surface, consistent with an in‐plane polarization configuration.

The coherence length extracted from the diffraction peak widths using the Scherrer equation (Figure 4a, right axis) remains below the nominal film thickness of 25 nm at all temperatures, as expected for a freestanding membrane with a high surface‐to‐volume ratio and finite crystalline coherence along the out‐of‐plane direction.

To further verify the unit‐cell orientation, reciprocal space maps of asymmetric (301) reflections, referenced to the bulk BTO tetragonal unit cell, were acquired at room temperature (Figure 4b,c). The diffracted intensity is centered close to the nominal positions expected for a tetragonal lattice oriented with the a‐axis perpendicular to the surface, confirming the validity of this structural model. Weaker intensity tails indicate a minority population of unit cells with different orientations, suggesting that the film is not monodomain but instead largely composed of in‐plane‐oriented tetragonal cells.

Taken together, the x‐ray diffraction results provide a framework to rationalize the temperature dependence of the electrical hysteresis observed in our devices. Our data support a structural picture at room temperature in which freestanding BTO membranes preferentially adopt unit‐cell orientations with their long axis, and thus their spontaneous polarization, lying parallel to the sample surface, and the BTO enters the tetragonal phase. This configuration naturally explains the absence of ferroelectric hysteresis in the electrical characteristics at room temperature, where an out‐of‐plane polarization component is weak or depolarized. In the present device geometry, where the freestanding BTO is directly interfaced with a metallic gate electrode and the semiconducting channel, this out‐of‐plane polarization component becomes sensitive to electrostatic boundary conditions, leading to enhanced depolarization and progressive suppression of the ferroelectric hysteresis (Figure 2b).

### 2.5 Temperature‐Dependent Output Characteristics

At low temperature (rhombohedral/orthorhombic phases), polarization directions include substantial projections along multiple crystallographic axes. Consequently, even if the energetically preferred configuration remains largely in‑plane, an out‑of‑plane component can be stabilized under reduced thermal activation and modified depolarization dynamics. In this regime, depolarization‑induced back‑switching is expected to start at film surfaces and propagate via domain‑wall motion [17, 36]. Suppressed domain‑wall mobility at low temperature increases coercive fields and can reduce back‑switching. This coincides with the pronounced memory window observed in the FET transfer curves at 10 K (Figure 3). Moreover, at low temperature the gate sweeps show counter‐clockwise hysteresis and independence of the sweep rate (Figure S2), signs for a ferroelectric nature [28, 37]. The hysteresis windows at low temperatures are more pronounced as trap states in the MoS_2_ channel and at the BTO/MoS_2_ interface are partially frozen, suppressing clockwise, trap‐dominated artifacts [38]. Increased coercivity at lower temperatures is also attributed to reduced domain wall mobility, as domain walls become increasingly pinned at low temperatures [39, 40] and reduced thermal activation energy [41].

**FIGURE 5:**
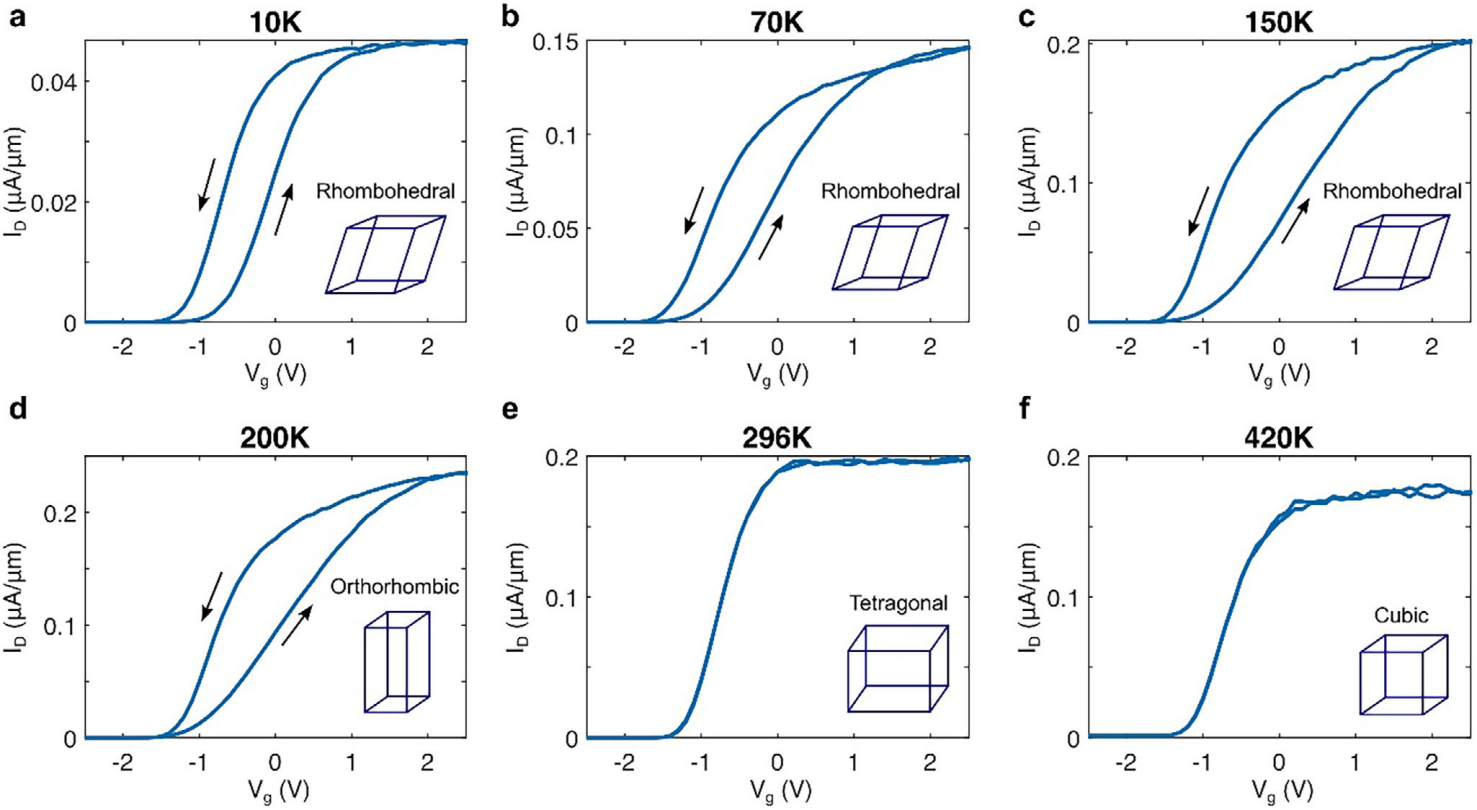
Temperature dependency of BTO/1L‐MoS_2_ FET. Temperature‐dependent transfer characteristics of a BTO/MoS_2_ ferroelectric field‐effect transistor displayed on linear scale, measured across six temperatures spanning the major structural phases of barium titanate: 10 K (rhombohedral phase), 70 K (rhombohedral phase), 150 K (rhombohedral phase, transition to orthorhombic phase), 200 K (orthorhombic phase), 296 K (tetragonal ferroelectric phase at room temperature), and 420 K (cubic paraelectric phase above the Curie temperature). The transfer curves reveal pronounced counterclockwise hysteresis loops at cryogenic temperatures, progressively diminishing hysteresis through intermediate temperatures, and near‐complete hysteresis collapse at 420 K where BaTiO_3_ transitions to the paraelectric regime (Curie temperature ∼403 K). V_DS_ = 0.5 V.

The XRD measurements therefore provide a structural landscape that is fully consistent with the temperature‐dependent electrical response of the BTO/MoS_2_ transistors. Importantly, the temperature scales at which inflections are observed in the XRD‐derived lattice parameter and coherence length (Figure 4a; ∼170 K, ∼300 K, and ∼380 K) closely coincide with changes in the hysteresis evolution of the FET shown in Figure 5. A maximum memory window is observed around the rhombohedral‐orthorombic phase transition around 150–200 K. Similar temperature‐dependent changes in ferroelectric hysteresis have been reported for BaTiO_3_ [42], where the coercive field and hysteresis losses vary strongly across structural phase transitions and show a maximum at 183 K. The memory window depends on both the ferroelectric coercive field and the kinetics of domain‐wall motion [43]. The maximum memory window observed here around 150–200 K may therefore result from enhanced electrically accessible polarization switching near the rhombohedral‐orthorhombic phase transition of BTO. While thin freestanding membranes may exhibit broadened or shifted transition temperatures compared to bulk BTO, this correlation supports a picture in which both thermally activated depolarization dynamics and phase‐dependent polarization anisotropy jointly govern the stabilization of out‐of‐plane ferroelectric coupling in freestanding BTO/MoS_2_ transistors. *SS* and on/off ratios do not change significantly from 10 K up to room temperature, at 420 K though the *SS* increases to 330 mV dec^−1^ and the on/off ratio decreases to 2 × 10^2^. All measurements were performed on a single device to enable a consistent analysis of the temperature‐dependent device physics without device‐to‐device variability.

Previous studies have demonstrated that interfacial screening charges naturally form at ferroelectric oxide/2D van der Waals interfaces and can strongly influence the measured hysteresis. In graphene and MoS_2_ devices gated by ferroelectric oxides, the dynamic response of these interfacial species to gate bias can over‑screen the ferroelectric polarization and even invert the hysteresis direction at elevated temperatures, whereas cooling suppresses their mobility and allows the intrinsic ferroelectric switching to dominate [16]. Defect‑induced interfacial charge traps can similarly compete with polarization doping and are also thermally activated [44, 45]. Our measurements in vacuum and after high‑temperature annealing reduce the density of adsorbates but cannot entirely eliminate such interfacial charges, so their temperature‑dependent dynamics likely contribute to the evolution of the hysteresis in BTO/MoS_2_ transistors alongside domain‑wall processes and phase transitions in BTO.

## 3 Conclusion

In summary, we have demonstrated thermally reconfigurable polarization coupling in freestanding BaTiO_3_ membranes integrated with monolayer MoS_2_ transistors. When directly transferred onto metallic gate electrodes, the BTO membrane functions at room temperature as an ultrahigh‐κ dielectric, enabling steep subthreshold swing and strong electrostatic control without ferroelectric hysteresis. Upon cooling, however, robust and stable counter‐clockwise hysteresis emerges, indicating the stabilization of an out‐of‐plane ferroelectric polarization component. Temperature‐dependent x‐ray diffraction reveals structural signatures consistent with the bulk phase sequence of BaTiO_3_ and confirms a predominantly in‐plane lattice orientation at room temperature, providing a structural framework for understanding the electrical response.

The combined electrical and structural results indicate that the interplay between phase‑dependent polarization anisotropy, electrostatic boundary conditions, residual depolarizing fields arising from imperfect screening, and the temperature‑dependent dynamics of interfacial screening charges and traps governs the stabilization of ferroelectric coupling in freestanding membranes. These findings establish freestanding perovskite oxides as multifunctional gate materials that can transition between high‐*κ* transistor operation and ferroelectric memory behavior depending on temperature. Beyond the specific BTO/MoS_2_ system, this work outlines design principles for engineering polarization orientation and depolarization control in oxide/2D heterostructures, opening pathways toward reconfigurable and phase‐adaptive electronic devices.

## 4 Materials and Methods

### 4.1 Electrode Deposition

Buried source‐drain‐gate electrodes were fabricated by mask‐less lithography (Microlight 3D SmartPrint UV), a 50 nm etch of the SiO_2_ layer (Glass etching cream Armour Etch) and subsequent thermal evaporation of 5 nm Cr (used as adhesion layer) and 45 nm Au onto a SiO_2_ (290 nm)/Si (p^++^) substrate, creating electrodes buried within the SiO_2_ layer, ideal for flake transfer. The method is described in more detail in Ref. [4].

### 4.2 BTO Growth, Delamination and Transfer

BTO (25 nm) films were grown epitaxially onto (001) STO substrates with a sacrificial layer of LSMO (15 nm) in between. Growth of both films was performed sequentially by pure oxygen (3.2 mbar) sputtering technique at high temperature (900°C) in vacuum. Afterward, a PPC solution (15 w.t.% in anisole) was spin–coated onto the grown structure and dried on a hotplate at 80°C for 10 min. After the structure cooled down, a piece of polydimethylsiloxane (Gel‐Film WF 4 × 6.0 mil by Gel‐Pack) was adhered to the LSMO/BTO and the stack was immersed in a diluted solution of 0.5 mL KI (3 mol/L) + 0.5 mL HCl (37%) and 10 mL deionized H_2_O at room temperature, dissolving the sacrificial LSMO layer over the course of 3 days and allowing the delamination of the BTO layer without damaging its properties.

The BTO is transferred onto a SiO_2_/Si substrate as follows. First, the BTO/PPC/PDMS is attached to the SiO_2_/Si substrate, with the BTO in contact with the substrate. The substrate is heated to 90°C and the PDMS is slowly retracted, leaving the BTO/PPC on the substrate. Now, the substrate is heated at 150°C for 5 min to increase the surface adhesion of the BTO film. As a last step, the PPC is dissolved by placing the substrate into acetone for 5 min.

From the SiO_2_/Si substrate, BTO flakes are selected and then transferred onto the pre‐fabricated electrodes via a dry PVC‐based stamping technique [46]. A piece of PVC food wrap (Riken Technos) is placed on top of a PDMS pyramid and used as a pick‐up layer. We follow the procedure disclosed in Ref. [20].

### 4.3 MoS_2_ Exfoliation and Transfer

MoS_2_ flakes were mechanically exfoliated from natural molybdenite mineral (Molly Hill Mine, Quebec, Canada) using Nitto tape (Nitto SPV 224) onto a PDMS substrate (Gel‐Film WF 4 × 6.0 mil by Gel‐Pack). Subsequently, chosen flakes were transferred using a dry‐transfer approach [24].

### 4.4 Electrical Characterization

All electrical measurements were performed in vacuum inside a commercial closed‐cycle refrigeration probe station (Lakeshore CRX‐6.5), equipped with a high‐temperature (HT) stage. The HT stage allows to thermally anneal the samples in vacuum at 500 K before characterization, while having the ability to cool down to 10 K during measurements. The temperature was adjusted with two temperature controllers (Lakeshore Model 336). To perform electrical transistor characterization a source‐meter unit (Keithley 2450) was used to measure drain current while applying a source‐drain voltage and two DC power supplies (Tenma 72–2715) back‐to‐back were used to apply gate bias.

### 4.5 Diffraction Measurements

θ–2θ scans were collected with Cu (K_α1_+K_α2_) radiation on a Bruker D8 Advance and a Panalytical X'Pert PRO Theta‐Theta diffractometer. The latter was equipped with: a Phenix low–temperature chamber by Oxford Cryosystems (for low temperature measurements between 15 and 300 K under vacuum) and an Anton Paar HTK 2000 chamber (for high temperature measurements between 300 and 473 K under N_2_ atmosphere).

Reciprocal space maps were collected at room temperature with Cu K_a1_ radiation on a Bruker D8 Discover 4‐circles diffractometer equipped with a two‐bounces Ge monochromator and a DECTRIS EIGER2 500 2D detector.

## Author Contributions

T.P. led sample patterning, device fabrication methodology and electrical measurements, as well as data analysis. F.M. and M.G.‐H. performed XRD measurements and data curation. V.R., C.L. and J.S. led perovskite growth and release. C.M., M.G.‐H. and A.C.‐G. led conceptualization, project administration, resources and supervision. T.P., C.M., M.G.‐H. and A.C.‐G. wrote the original draft. All authors contributed to the review and editing of the manuscript.

## Conflicts of Interest

The authors declare no conflicts of interest.

## Supporting information




**Supporting File**: advs77639‐sup‐0001‐SuppMat.pdf.

## Data Availability

The data that support the findings of this study are available from the corresponding authors upon reasonable request.
