# Supplementary material for: Mechanisms of Temperature‐Dependent Hysteresis in Freestanding BaTiO3/MoS2 Heterostructures
Source: Adv Sci (Weinh). 2026 Sep 8:e77639. Online ahead of print. doi: 10.1002/advs.77639 (PMC13554489; doi:10.1002/advs.77639)
Supplement: Supplementary file 1 [file ADVS-9999-e77639-s001.pdf]

Supporting Information

Mechanisms of temperature-dependent hysteresis in freestanding $BaTiO_3/MoS_2$ heterostructures

Thomas Pucher[1], Federico Mompeán[1,2], Víctor Rouco[2,3], Carlos León[2,3], Jacobo Santamaría[2,3], Carmen Munuera[1,2], Mar García-Hernández[1,2], Andres Castellanos-Gomez[1,2]**

[1]2D Foundry Research Group. Instituto de Ciencia de Materiales de Madrid (ICMM-CSIC), Madrid, E-28049, Spain.

[2] Unidad Asociada UCM/CSIC, "Laboratorio De Heteroestructuras con Aplicación En Spintrónica", Madrid, Spain

[3] GFMC, Departamento de Física de Materiales, Facultad de Física, Universidad Complutense, Madrid, Spain

*corresponding authors thomas.pucher@csic.es , andres.castellanos@csic.es

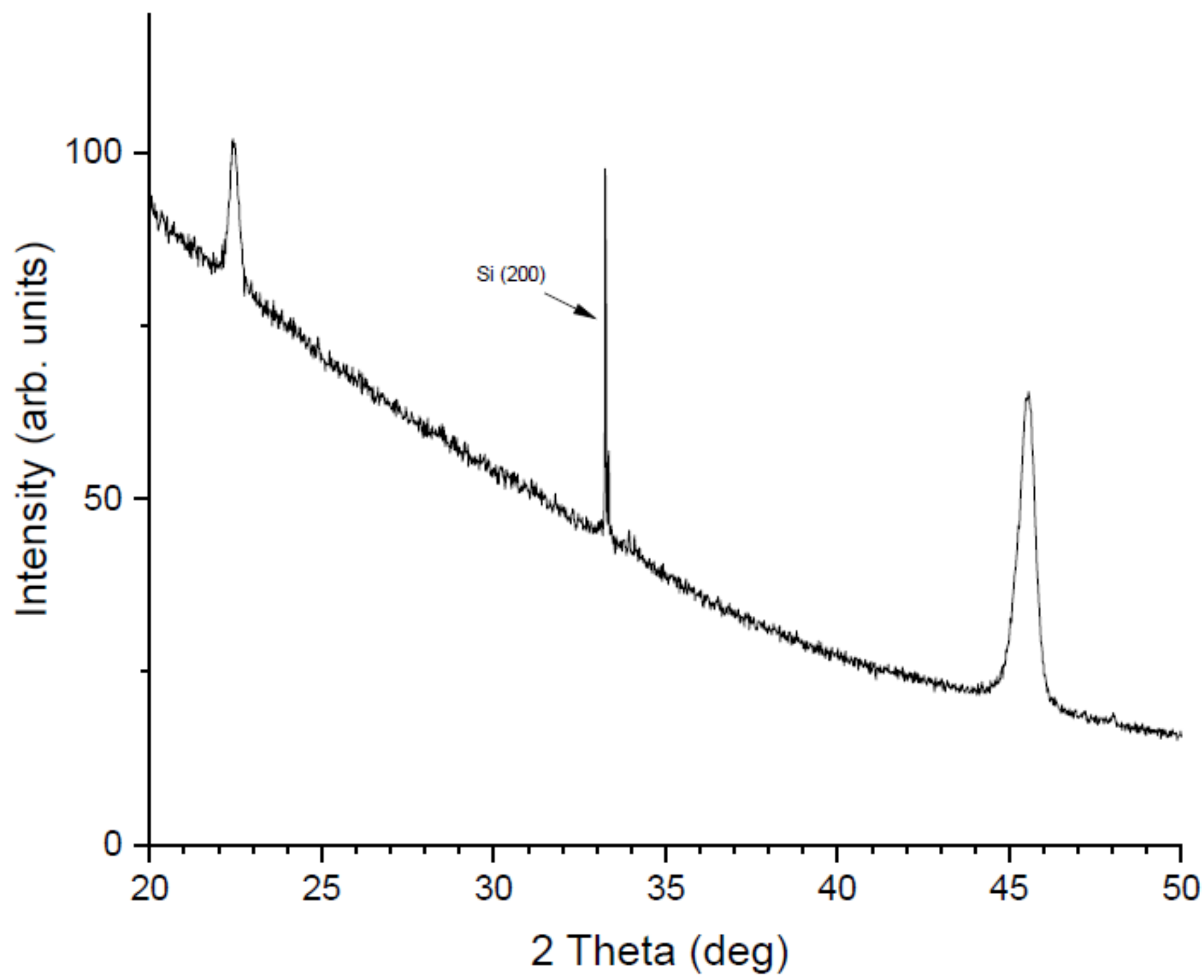

Figure S1. shows θ-2θ scans at room temperature and ambient atmosphere recorded with the sample substrate placed on a glass sample holder. The most prominent features in the 2θ = 20 to 50 degree range are the two peaks from the Si (100) substrate corresponding to the "forbidden" reflection by Si (200) planes brought about by Renninger multiple scattering from the Cu ($K\alpha_1$+$K\alpha_2$) lines. These narrow peaks serve as reference for the instrumental resolution and enable us to compensate for the z-error incurred while aligning the sample on a powder diffractometer. The interesting regions for the BTO membrane analysis are the broad peaks found near 2θ = 22.4 and 45.4 degrees. By reference to bulk BTO in the tetragonal phase (Space Group P4mn with a = b = 0.3994 nm and c = 0.4038 nm) these peaks fall respectively in the positions corresponding to

(100) and (200) reflections. A similar analysis has been done with the θ-2θ scans collected as a function of temperature.

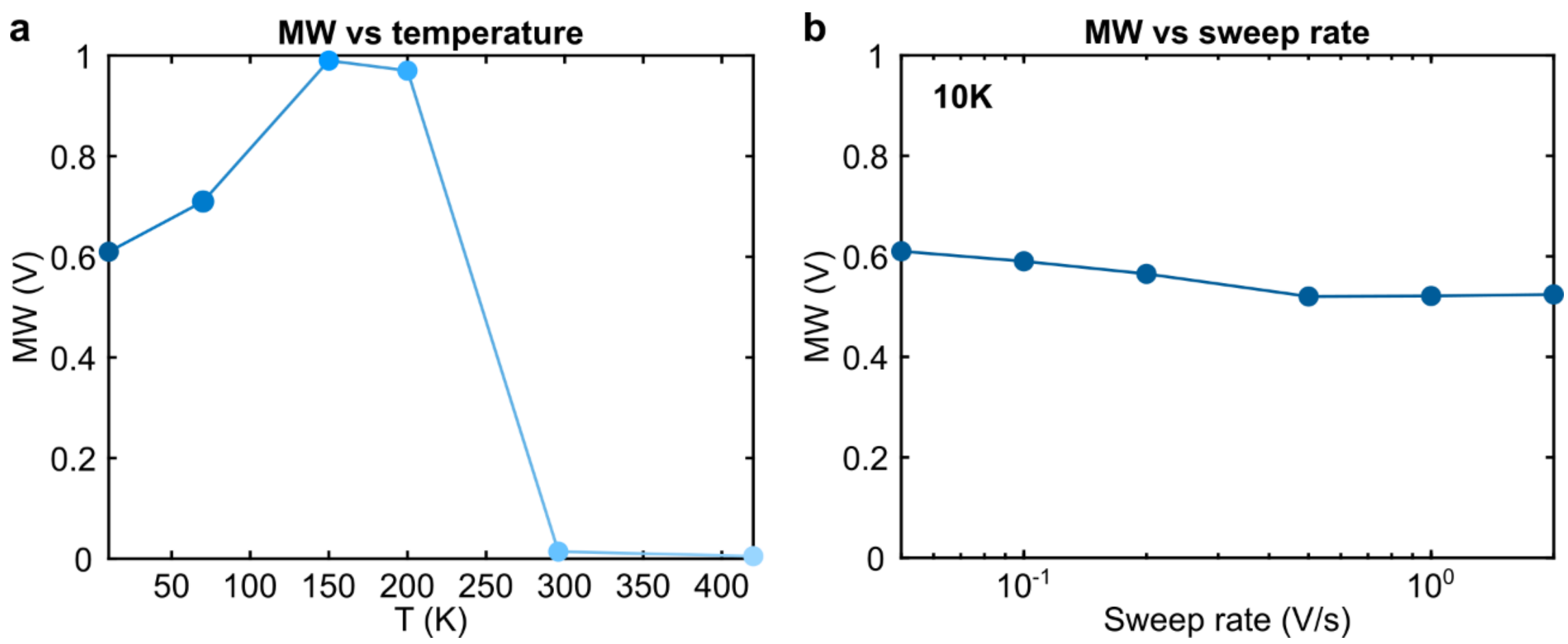

Figure S2. (a) Memory window (MW), extracted as the difference between the threshold voltages of the forward and backward gate-voltage sweeps, as a function of temperature for the BTO/1L-MoS_2 transistor. The memory window remains pronounced throughout the low-temperature ferroelectric phases of $BaTiO_3$, reaching a maximum around 150–200 K, and collapses upon approaching room temperature and the paraelectric regime, consistent with the temperature-dependent transfer characteristics shown in Figure 5. (b) Memory window measured at 10 K as a function of gate-voltage sweep rate. The weak dependence of the memory window on sweep rate over nearly two orders of magnitude indicates that the observed hysteresis is dominated by ferroelectric polarization switching rather than charge trapping processes, which typically exhibit a stronger time dependence. Measurements were performed at $V_{DS} = 0.5\text{V}$.
